# Methylation of Estrogen Receptor 1 Gene in the Paraspinal Muscles of Girls with Idiopathic Scoliosis and Its Association with Disease Severity

**DOI:** 10.3390/genes12060790

**Published:** 2021-05-21

**Authors:** Piotr Janusz, Małgorzata Chmielewska, Mirosław Andrusiewicz, Małgorzata Kotwicka, Tomasz Kotwicki

**Affiliations:** 1 Department of Spine Disorders and Pediatric Orthopedics, Poznan University of Medical Sciences, 28 Czerwca 1956 r. Street 135/147, 61-545 Poznan, Poland; pjanusz@ump.edu.pl (P.J.); kotwicki@ump.edu.pl (T.K.); 2 Chair and Department of Cell Biology, Poznan University of Medical Sciences, Rokietnicka 5D, 60-806 Poznan, Poland; andrus@ump.edu.pl (M.A.); mkotwic@ump.edu.pl (M.K.)

**Keywords:** spinal curvatures, scoliosis, idiopathic, DNA methylation, pyrosequencing, estrogen receptor 1, ESR1, scoliosis progression, adolescent idiopathic scoliosis

## Abstract

Idiopathic scoliosis (IS) is a multifactorial disease with epigenetic modifications. Tissue dependent and differentially methylated regions (T-DMRs) may regulate tissue-specific expression of the estrogen receptor 1 gene (*ESR1*). This study aimed to analyze methylation levels within T-DMR1 and T-DMR2 and its concatenation with ESR1 expression of IS patients. The study involved 87 tissue samples (deep paravertebral muscles, both on the convex and the concave side of the curve, and from back superficial muscles) from 29 girls who underwent an operation due to IS. Patient subgroups were analyzed according to Cobb angle ≤70° vs. >70°. Methylation was significantly higher in the superficial muscles than in deep paravertebral muscles in half of the T-DMR1 CpGs and all T-DMR2 CpGs. The methylation level correlated with *ESR1* expression level on the concave, but not convex, side of the curvature in a majority of the T-DMR2 CpGs. The T-DMR2 methylation level in the deep paravertebral muscles on the curvature’s concave side was significantly lower in patients with a Cobb angle ≤70° in four CpGs. DNA methylation of the T-DMRs is specific to muscle tissue location and may be related to *ESR1* expression regulation. Additionally, the difference in T-DMR2 methylation may be associated with IS severity.

## 1. Introduction

The most common spine disorder in adolescents is idiopathic scoliosis (IS), affecting 1–3% of the population. It is a structural, three-dimensional spinal deformity characterized by lateral curvature of the spine, impaired kyphosis or lordosis, and vertebral rotation with a rib hump [1]. IS is a highly heterogeneous condition, with some patients having a rapidly progressive presentation, resulting in severe curves, and others progressing slowly to mild or moderate curves [2]. Progressive scoliosis may result in cosmetic deformity, back pain, and functional deficits as well as psychological problems and impaired social interactions. Severe curvatures are associated with cardiac dysfunction and pulmonary constraints [3,4,5]. Currently, clinical or radiological criteria cannot adequately predict which children who are diagnosed with mild disease may ultimately undergo subsequent curve progression that requires surgical intervention [6]. Identifying patients at risk of scoliosis, or those at risk of curve progression, is essential for early, appropriate treatment [1,7].

Despite the high prevalence of IS, its etiology remains poorly understood [8]. IS is considered a multifactorial disease with genetic susceptibilities [9]. Many candidate genes potentially associated with IS have been described in family linkage studies, single nucleotide polymorphisms association studies, and genome-wide association studies [8,10,11,12]. Results from these suggest IS is a complex genetic disorder [6]. It was postulated that genetic factors are more important in the occurrence of IS while environmental factors have a more significant impact on disease progression [13]. An epigenetic link between genetic and environmental factors may be involved in IS etiopathogenesis [14]. As a new area of research, only a few publications concerning the impact of DNA methylation on IS have been published [15,16,17,18,19]. However, none of these studies have evaluated this mechanism in paraspinal muscle tissues.

Due to the gender-related distribution of idiopathic scoliosis, the role of estrogen hormones in IS occurrence and progression has been suggested [20,21]. Previous studies have reported the effect of estrogens on skeletal muscles, in which the mRNA and protein expression of estrogen receptor 1 and 2 (*ESR1*, *ESR2*) has been demonstrated [22,23,24]. *ESR1* and *ESR2* expression was confirmed in the superficial and deep paravertebral muscles of patients with IS. Moreover, expression of *PELP1* (proline-, glutamic acid-, and leucine-rich protein) was significantly higher in the deep back muscles compared to superficial muscles. This protein participates in estrogen-induced signal transduction pathways. Additionally, *PELP1* expression level was correlated with both the Cobb angle value and *ESR1* expression [25].

DNA methylation is one of the most well-characterized epigenetic modifications. Methylation of CpG islands (CGI), located near the transcription start site of gene promoter and regulatory regions, is associated with altered gene expression [26,27]. Methylation at the gene promoter inhibits recognition and binding of transcription factors. This leads to the recruitment of proteins binding to methylated CpG dinucleotides which, in turn, interact with transcription repressors and activate chromatin condensation by recruiting histone deacetylases. As a result, DNA methylation in CpG site-rich regions, found in close proximity to the promoter region, is thought to play an essential role in gene silencing [28]. It has been indicated that a different methylation status is characteristic for particular types of tissues or the development phase [29]. Although the CpG islands in intragenic and regulatory regions of genes may display a tissue-dependent and differentially methylated region pattern [30], CGIs associated with transcription start sites rarely show tissue-specific patterns of methylation [31].

**Figure 1:**
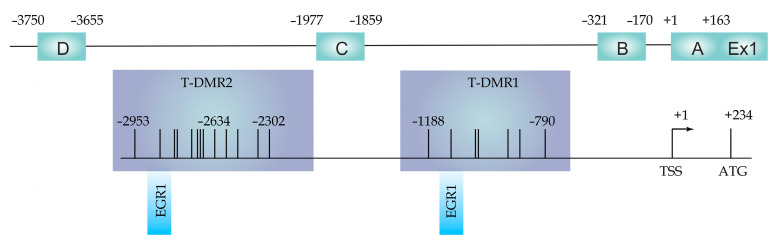
*ESR1* promoters (A-D) and T-DMR1, and T-DMR2 regions localization with respect to transcription start site (TSS) and translation start codon (ATG). EGR1 indicates transcription factor binding sites. Adapted from [34].

It was shown that in the case of ESR1, the level of methylation within the promoter is cell-specific [32]. Analysis of the C promoter (Figure 1) in in vitro study indicated that demethylation of this region is responsible for the increased expression of ESR1 [33]. The relationship between regulatory regions methylation of ESR1 and its expression was suggested. It has been reported that ESR1 has tissue-dependent and differentially methylated regions (T-DMRs; Figure 1), which are associated with tissue-specific gene expression [34]. A previous study showed that methylation at T-DMR1 and T-DMR2 is correlated with decreased ESR1 expression in the placenta and skin tissue but not in mammary glands and the endometrium [34]. Maekawa et al. suggested that ESR1 expression is tissue-specific and regulated by DNA methylation at T-DMR1 rather than by DNA methylation at the promoter region [34]. Thus, changes in ESR1 mRNA expression may not correspond with methylation of the ESR1 promoter. Moreover, it was indicated that, in the case of some breast cancer tissues, ESR1 expression might be modulated not only by DNA methylation at T-DMRs and promoter regions but also by different mechanisms that require clarification in future studies [34].

Taking into consideration that methylation level alterations among patients with different IS phenotypes may be associated with susceptibility to disease or disease progression, we therefore analyzed T-DMR1 and T-DMR2 methylation status. Subsequently, the expression level of *ESR1* in the superficial and paraspinal muscles on the convex and concave side of the IS curvature was analyzed and evaluated in relation to methylation status.

## 2. Results

### 2.1. Patient Characteristics

The study group consisted of 29 female IS patients (age at surgery: 12.1–17.9 years, mean age: 14.5 ± 1.5 years). The Cobb angle ranged from 52° to 115°, with a mean of 77.4 ± 16.1°. The mean age, number of curvatures, and Risser sign value did not differ significantly between the subgroups of patients with Cobb angles ≤70° and ≥70° (14.5 ± 1.3 vs. 14.7 ± 1.7, *p* = 0.9; 3 single: 7 double vs. 8 single: 11 double, *p* = 0.7; Me = 4 vs. Me = 4, *p* = 0.7, respectively). The mean Cobb angle value of patients with a Cobb angle ≤70° and those ≥70° was 61.1° ± 6° and 86° ± 12.7°, respectively.

### 2.2. DNA Methylation at the ESR1 T-DMR1 and T-DMR2

**Figure 2:**
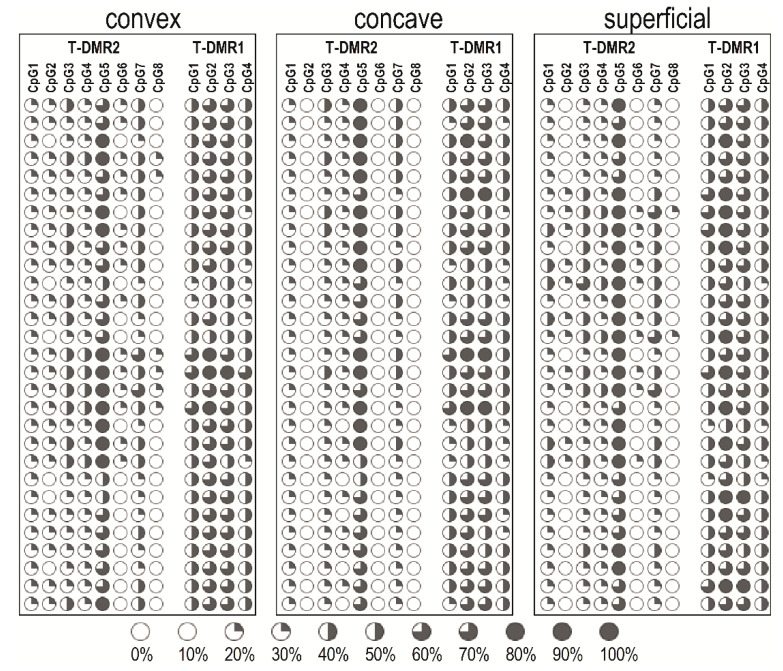
Dot plot of *ESR1* T-DMR1 and T-DMR2 regions methylation pattern of individual patients.

The methylation pattern within T-DMR1 and T-DMR2 of individual patients is shown in Figure 2.

**Figure 3:**
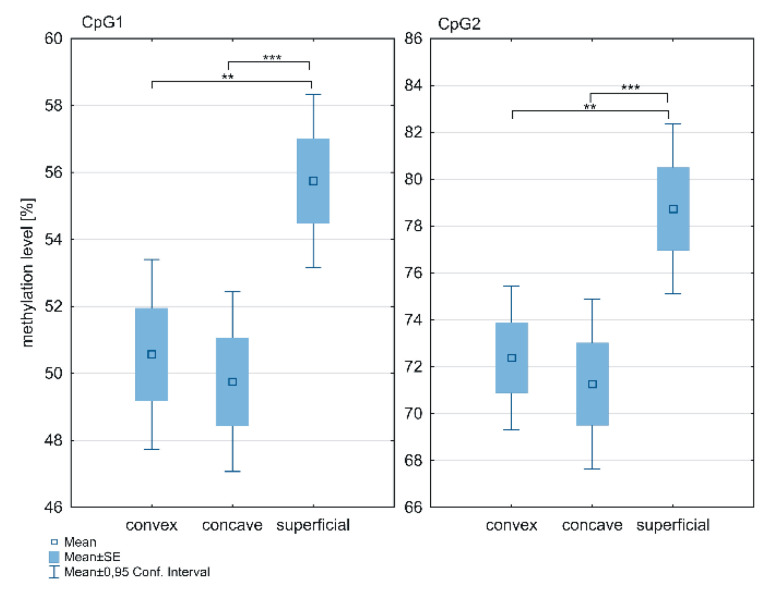
DNA methylation level within *ESR1* T-DMR1 region in deep paravertebral muscles and superficial muscles; ** *p* < 0.01, *** *p* < 0.001.

The methylation level within the *ESR1* T-DMR1 region was significantly higher in the superficial muscle compared to the deep paravertebral muscles at the CpG1 (*p* = 0.0001; Figure 3; Supplementary Table S1) and CpG2 sites (*p* < 0.0001; Figure 3; Supplementary Table S1). The methylation level was significantly higher in the superficial muscle compared to both, the concave (*p* < 0.05; Figure 3; Supplementary Table S1) and convex side of the curvature (*p* < 0.05; Figure 3; Supplementary Table S1). Moreover, in the deep paravertebral muscles, methylation was decreased on the concave side in contrast to the convex side of the curvature. However, the difference was not statistically significant (Supplementary Table S1).

**Figure 4:**
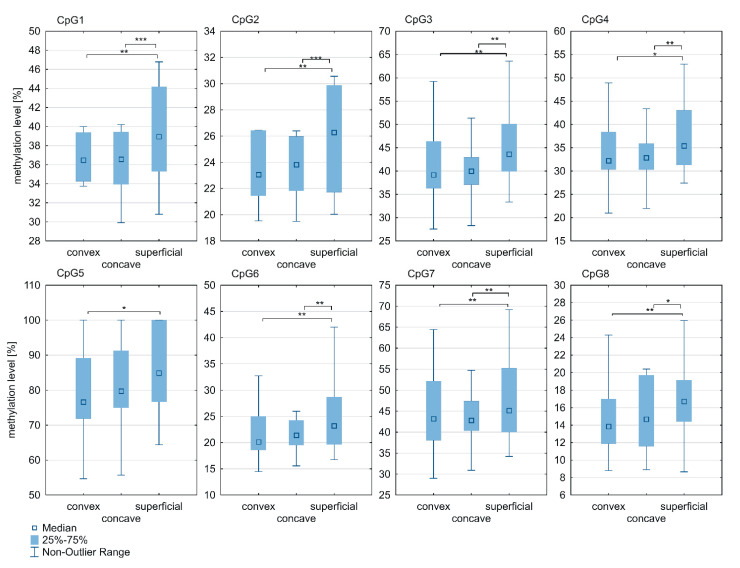
DNA methylation level within *ESR1* T-DMR2 region in deep paravertebral muscles and superficial muscles; * *p* < 0.05, ** *p* < 0.01, *** *p* < 0.001.

Significant differences in methylation levels of all CpG sites within the *ESR1* T-DMR2 region between superficial and deep paravertebral muscles were observed (*p* < 0.05; Figure 4; Supplementary Table S1). Methylation was found to be significantly higher in the superficial muscle versus the concave (at CpG sites 1–4 and 6–8; *p* < 0.05; Figure 4; Supplementary Table S1) and convex side of the curvature (at all CpG sites; *p* < 0.05; Figure 4; Supplementary Table S1). In contrast to the *ESR1* T-DMR1 region, the methylation level within the T-DMR2 region in the deep paravertebral muscles was lower on the convex side of the curvature in seven of eight CpGs compared to the concave side. However, the difference was not statistically significant (Figure 4; Supplementary Table S1).

### 2.3. Correlation between ESR1 Methylation Levels and Relative Expression of the ESR1 Gene

The ESR1 relative expression did not differ significantly between the deep paravertebral muscles on both, convex and concave side, and superficial muscles (*p* > 0.05; Supplementary Figure S1).

**Figure 5:**
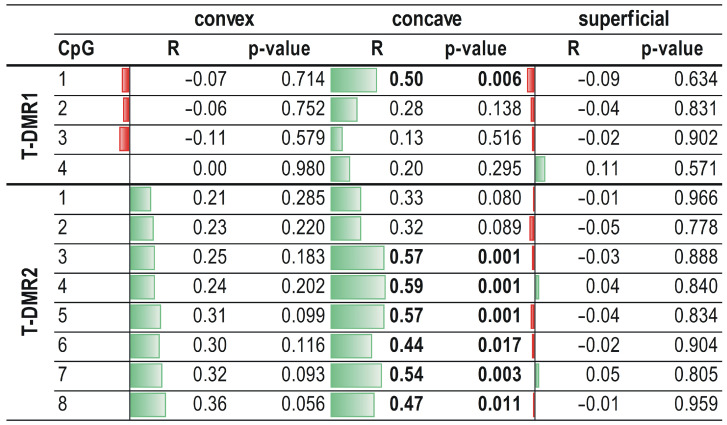
Correlation between *ESR1* expression and methylation level within T-DMR1 and T-DMR2 regions in deep paravertebral muscles and superficial muscles. R—Spearman rank correlation coefficient.

On the concave side of the curvature, a significant, moderate, and positive correlation was observed between *ESR1* mRNA expression and methylation level at the CpG1 dinucleotide in the T-DMR1 region and at six CpG sites (CpG2-CpG8) in the T-DMR2 region (R ranged from 0.44 to 0.59; *p* < 0.05; Figure 5). No correlation between *ESR1* expression and methylation level within the T-DMR1 and T-DMR2 regions was found either in the superficial muscle or on the convex side of thoracic scoliosis (*p* > 0.05; Figure 5; Supplementary Figures S2 and S3).

### 2.4. Association between Methylation Status of ESR1 and Cobb Angle

**Figure 6:**
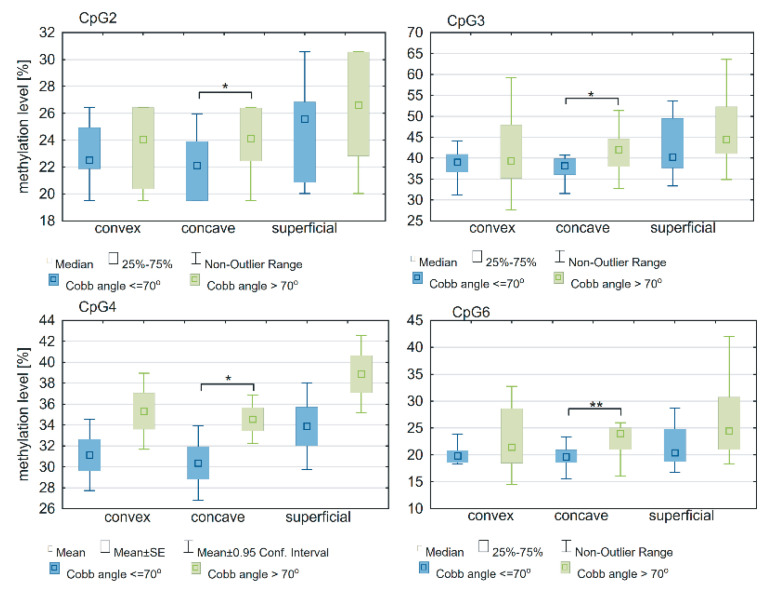
DNA methylation level within *ESR1* T-DMR2 region in deep paravertebral muscles and superficial muscles in patients with Cobb angles ≤70° and >70°; * *p* < 0.05, ** *p* < 0.01.

In the deep paravertebral muscle, the methylation level within the *ESR1* T-DMR2 region on the concave side of the curvature was significantly different between groups of patients with a Cobb angle >70° or ≤r0° at four CpG sites: CpG2 (*p* = 0.02; Figure 6; Supplementary Table S2), CpG3 (*p* = 0.04; Figure 6; Supplementary Table S2), CpG4 (*p* = 0.04; Figure 6; Supplementary Table S2) and CpG 6 (*p* = 0.005; Figure 6; Supplementary Table S2). There was no difference in the *ESR1* T-DMR1 region methylation level between groups of patients with a Cobb angle ≤70° or >70° (*p* > 0.05; Supplementary Table S2). No differences were observed in T-DMR1 methylation levels between groups of patients with Cobb angles ≤70° and >70° (*p* > 0.05; Supplementary Table S2).

No correlation was found between T-DMR1 region methylation level and Cobb angle value in either the superficial or deep paravertebral muscle tissues (r ranged from 0.02 to 0.23; *p* > 0.05). Examining the concave side of thoracic scoliosis, a significant, moderate and positive correlation between T-DMR2 methylation and Cobb angle was observed at CpG2 (R = 0.44; *p* = 0.02) and CpG6 (r = 0.5; *p* = 0.005). There was no significant correlation between T-DMR2 methylation and Cobb angle in the superficial muscles (CpG2 and CpG4-CpG8, R ranged from 0.03 to 0.27 (*p* > 0.05); CpG1 and CpG3, r ranged from 0.12 to 0.14 (*p* > 0.05)) or in the deep paravertebral muscles on the convex side of the curvature (CpG1, CpG2, CpG6 and CpG8, R ranged from 0.03 to 0.24 (*p* > 0.05); CpG3-CpG5 and CpG7, r ranged from 0.07 to 0.26 (*p* > 0.05)).

## 3. Discussion

Although the history of IS has been thoroughly described and treatment methods are established, the exact etiology and pathology have yet to be elucidated [1,8,35]. IS has been extensively analyzed with respect to susceptibility to scoliosis development and curvature progression, with various theories concerning IS etiology suggested. Recently, genetic studies revealed an important association between DNA polymorphisms and disease susceptibility and severity [36,37,38]. Despite promising results, these studies did not provide insight to any IS predisposition nor provide a molecular explanation of the disease. It has become increasingly apparent that many diseases are likely the result of interactions between genes and the environment [2]. According to Grauers et al., 38% of the variance in the liability of IS development is due to additive genetic effects and 62% to unique environmental effects [39]. Thus, one of the most interesting hypotheses regarding IS etiology is the linkage of genetic susceptibilities to environmental factors.

Several epigenetic studies concerning the etiopathogenesis of IS have been conducted. As of now, five of them described DNA methylation as an epigenetic mechanism associated with IS. Mao et al. evaluated methylation levels of the cartilage oligomeric matrix protein gene. They found that hypermethylation of the gene promoter correlated with adolescent idiopathic scoliosis (AIS) curve severity [16]. Shi et al. published two studies concerning DNA methylation in AIS and revealed an association of paired-like homeodomain 1 and protocadherin-10 gene methylation with IS susceptibility and curvature severity [17,18]. Meng et al. conducted analysis of the whole-genome methylation in two pairs of twins. They found an association between methylation levels at site cg01374129 and curve severity [19]. Liu et al. also performed whole-genome methylation analysis in a pair of twins. They discovered several signaling pathways potentially associated with AIS and a significantly higher methylated region in chromosome 15 of the AIS group [15]. All mentioned studies concerning DNA methylation were performed with peripheral blood samples. In the search for the molecular explanation of IS, we analyzed the local molecular predisposition to IS occurrence or progression at the apex of the curvature. We focused on paraspinal muscles as a possible target tissue for locally acting factors as these muscles play a key role in controlling spinal stability [2]. There is a hypothesis that dysfunctional paraspinal muscles may contribute the development of the scoliotic curve [2,40]. Additionally, reports have described functional and histological differences in the paraspinal muscles between the convex and the concave sides of the curve in IS patients [41,42].

An interesting feature of IS is the correlation of disease severity with gender, especially after puberty. The female/male ratio in mild scoliosis is reported to be 1.4/1, while in severe scoliosis, it is estimated to be 8.4/1 [13,20,43]. This shift suggests a relationship between sex hormones with a clinical manifestation of IS [43,44]. As *ESR1* and *ESR2* are known to mediate the effects of estrogens, they became the subject of genetic studies concerning DNA polymorphisms of *ESR1* and *ESR2* in IS. Although early studies were promising, they failed to be replicable in subsequent studies [45,46]. A recent cross-sectional study revealed that some *ESR1* and *ESR2* variants were associated with the occurrence risk of idiopathic scoliosis [12]. Meta-analysis performed by Sobhan et al. suggested that *ESR1* polymorphisms rs9340799 and rs2234693 are not related to the risk of IS occurrence. However, rs9340799 may be associated with the risk of developing AIS among the Asian population [47]. Due to the unknown role of estrogens and their receptors in IS etiology, we evaluated the gene methylation status of estrogen receptors in IS.

In our study, we found differences in methylation levels between the deep paravertebral muscles (*m. longissimus*) and the superficial muscles (*m. trapezius*) in two CpGs of T-DMR1 and in all CpGs of T-DMR2. We consider the superficial muscle as a control, due to its distance from deformation, anatomical borders (fascia layers) between it and the deep muscles, different embryogenesis and function from the deep muscles, and a disparate nerve supply. Thus, this observed difference in methylation supports the theory of distinctive methylation patterns depending on localization in the same tissue type. Slieker et al. identified, using genome-wide DNA methylation data, that there are T-DMRs in CpG-poor regions such as CGI shores or distal promoters, which are associated with aberrant transcription. Interestingly, the authors observed interindividual variation of DNA methylation for more than 8000 CpGs in the skeletal muscle tissue and within-individual methylation differences between muscle and blood tissues for over 2000 CpGs [48]. Therefore, the interpretation of methylation patterns for tissues representing cellular heterogeneity, such as skeletal muscle, is particularly complex. It is also a challenge in comparative tissue research [48,49,50]. According to Maekawa et al., *ESR1* has tissue-dependent and differentially methylated regions (T-DMRs), which are associated with tissue-specific gene expression [34].

Our results did not reveal a difference in DNA methylation between the concave and convex side of paravertebral muscles when all patients were considered together. However, we found a difference in DNA methylation between patients with a Cobb angle ≤70° and >70° in the T-DMR2 region at the concave side of the curvature. Moreover, in CpG2 and CpG6 in the T-DMR2 region, the level of methylation at the concave side of the curvature correlated with the Cobb angle value. According to the abovementioned studies concerning the differences between paraspinal muscles at the apex of the curvature, this side was significant in relation to the etiopathogenesis of IS [51,52,53]. The absence of this correlation in the superficial and paravertebral muscles on the convex side in any of the evaluated CpGs also supports the importance of the concave side of the curvature in predisposition to IS progression. Thus, we interpret these results as a lack of association of *ESR1* methylation with a predisposition to IS development and consider methylation status as an IS phenotype modifier rather than the direct molecular background. These results are in line with the opinion of Cheung et al. who state that some factors may contribute to curve progression while others contribute to curve initiation [54]. According to Leboeuf et al., estrogens may be considered contributing factors in the progression of scoliosis [55]. Our results support this hypothesis from an epigenetic point of view.

When debating on changes of the paravertebral muscles at the level of the concave side of the curvature, there exists a dilemma regarding the primary versus secondary nature due to impaired spine biomechanics (asymmetrical loading). It is possible that the difference in methylation may contribute to some asymmetry in muscle function and promote curvature progression. On the other hand, the presented results may be a consequence of exposure to different local mechanical conditions due to asymmetric loading or other unknown factors. In our opinion, the results of this study most likely reveal primary changes. The impact of asymmetrical loading or other factors on the methylation level should be detectable in all evaluated CpGs, not only in specific ones. To evaluate the direct impact of DNA methylation on IS progression, two patient subgroups were distinguished. The patients were divided according to disease severity and its possible impact on the patient health [35,56,57]. The skeletally mature patients with a Cobb angle between 50° and 70° require surgical scoliosis correction to avoid further curvature deterioration into adulthood [35,56,57]. Whereas severe curvatures can negatively impact patient health including outcomes such as decreased lung function, cardiac function, back pain, and degenerative spine disease [3,5,57]. There is no solid threshold for Cobb angle value when curvature significantly impacts the patients’ health. Studies concerning surgical treatment of IS classify scoliosis as a severe when a curvature exceeds 70° in Cobb angle [58,59,60]. Thus, we used this value to categorize study subgroups.

Contrary to previous studies, we observed a positive correlation between *ESR1* expression and methylation level within regulatory regions. Maekawa et al. showed that *ESR1* expression was tissue specific and downregulated by DNA methylation at T-DMRs in normal tissues but not always in breast cancer. They have also evaluated the expression level of different *ESR1* variants and suggested that there is interplay between DNA methylation of T-DMRs and regions around upstream exons [34]. Our result, different from those of Maekawa [34], may be explained by the fact that we examined all transcription variants in one quantitative reaction. Additionally, we did not evaluate methylation of promoter regions but only both T-DMRs. Moreover, it is indicated that T-DMR methylation may modulate the availability of DNA sequences for methylation-dependent transcription factors [61]. Those findings are in line with results presented by Maekawa et al., who identified that EGR1 (early growth response protein 1) may be the potential transcription factor that binds to the T-DMRs and, as a result, upregulates *ESR1* expression [34]. It has also been suggested that there are T-DMRs negatively and positively correlated with gene expression depending on genomic localization [61].

Direct comparison of our results with other published studies concerning methylation of DNA in IS was challenging due to different tissue samples used for evaluation. Peripheral blood is a very good source of DNA when polymorphisms are considered. However, the methylation level obtained from the blood will only show the methylation level of whole DNA without specific local disturbances. Thus, a strong point of this study was the evaluation of tissues at the center of the pathology, thereby bringing forth new facts about the impact of *ESR1* DNA methylation on the IS phenotype.

Our study reveals new aspects concerning IS etiopathogenesis. It develops a further explanation of why some IS progress more often than others. A better understanding of the pathology improves the diagnosis and treatment methods. However, the direct clinical implications of this study are limited. Genetic studies aim to develop a test that may help distinguish the prognosis between patients with severe disease from a more benign condition. We hope that further studies beyond our results can be useful in the development of such a test.

The main limitation of our study is the lack of a healthy control group. It was impossible to obtain paraspinal muscles samples from healthy, age-matched females. We considered harvesting muscle samples from patients who have undergone a surgery due to degenerative spine disease. However, the vast difference in patient age and the muscle atrophy due to long-lasting degenerative spine disease may induce unknown methylation changes. This would be possible introduction of bias rather than a reliable evaluation of methylation impact on the etiology of IS. Another limitation in this study was sample size. However, it is comparable with other studies evaluating DNA methylation in IS even though the other studies were performed on blood samples [15,16,17,18,19].

## 4. Materials and Methods

### 4.1. Patient Population

The study group consisted of 29 girls who underwent an operation due to IS between January 2017 and December 2019 at the Department of Spine Disorders and Pediatric Orthopedics in Poznan University Hospital. All patients met the following inclusion criteria: (1) confirmed diagnosis of IS (other backgrounds of scoliosis were excluded); (2) no coexisting genetic, neurological, or orthopedic disorders; (3) thoracic location of the main curvature; (4) surgical treatment with posterior spinal instrumentation and fusion. All patients underwent clinical and radiological examinations, including long-cassette standing X-rays taken prior to surgery. Number, localization, and curvature size (Cobb angle) was measured [62]. Skeletal maturity was assessed by the Risser sign [63]. One experienced spine surgeon performed all measurements. Patients were divided into two subgroups according to final disease severity at skeletal maturity: scoliosis of equal to or less than 70° vs. greater than 70° as measured with the Cobb angle. The first subgroup (Cobb ≤ 70°) consisted of 10 patients without a major risk of significant impact on cardio-pulmonary function in adulthood. The second subgroup (Cobb > 70°) consisted of 19 patients with severely progressive IS that possibly may impact cardio-pulmonary function.

### 4.2. Tissue Samples

During surgery, 1 cm^3^ muscle tissue fragments were obtained from one deep paravertebral muscle (*m. longissimus thoracic*) on the convex and concave side of the curvature as well as from one superficial muscle (*m. trapezius*). Samples were stored at −80 °C in tubes containing nucleic acid preservation solution (Novazym, cat no. ST01; Poznan, Poland).

### 4.3. Genomic DNA Methylation Analysis

#### 4.3.1. Genomic DNA Isolation and Bisulfite Conversion

Total genomic DNA was extracted using a silica matrix column kit (Zymo Research, cat no. D4069; Irvine, CA, USA) with a modified protocol. In short, 25 mg of tissue samples ground in liquid nitrogen were incubated overnight at 55 °C with proteinase K. The lysate was then centrifuged (12,000× *g*, 1 min, room temperature). Next, the procedure followed the isolation according to manufacturer’s protocol. The gDNA quantity, purity, and integrity were assessed both spectrophotometrically and electrophoretically. One microgram of gDNA was bisulfite converted using an EZ DNA Methylation™ Kit (Zymo Research, cat no. D5002; Irvine, CA, USA) according to the manufacturer’s protocol.

#### 4.3.2. Polymerase Chain Reaction and Pyrosequencing Analysis

Bisulfite converted DNA served as the template for polymerase chain reaction (PCR) followed by pyrosequencing (PSQ). The primers for PCR and PSQ reactions were designed using PyroMark Assay Design software (version 2.0.1.15; Qiagen; Hilden, Germany). The input DNA sequences corresponded to the T-DMR1 and T-DMR2 regions of the *ESR1* gene (https://www.ncbi.nlm.nih.gov (accessed on 5 March 2019); GenBank N°: NG_008493.2). Sequencing, forward, and biotinylated reverse primers are presented in Table 1.

Polymerase chain reactions were performed using ZymoTaq^TM^ PreMix (Zymo Research; cat no. E2004; Irvine, CA, USA) designed for the amplification of bisulfite-treated DNA. Reaction mixture components, concentrations, and thermal profile is presented in Table 2. Two microliters of the product were separated using a standard 2% agarose gel and compared to molecular mass marker (Novazym, cat no. MA1000-03; Poznan, Poland).

PSQ analysis was performed using the PyroMark Q48 instrument (Qiagen; Hilden, Germany) according to CpG assays designed with Pyromark Q48 Autoprep 2.4.2 software (Qiagen; Hilden, Germany). Analysis of 4 and 8 CpG sites for T-DMR1 and T-DMR2, respectively, were performed (internal sodium bisulfite treatment quality control was included in each reaction). The methylation level was quantified using Pyromark Q48 Autoprep 2.4.2 software and expressed as a percentage ratio of methylated to non-methylated dinucleotides.

### 4.4. Analysis of ESR1 mRNA Expression

#### 4.4.1. Total RNA Isolation and Reverse Transcription

Total cellular RNA was extracted using Renozol (GenoPlast Biochemicals, cat no. BNGPB1100-2; Rokocin, Poland) and Direct-zol RNA Miniprep Kit (Zymo Research, cat no. R2052; Irvine, CA, USA) following the manufacturer’s protocol. RNA quantity and purity were assessed similarly to gDNA. RNA integrity was evaluated with 18S and 28S ribosomal RNA using 1% standard denaturing agarose gel electrophoresis.

Reverse transcription reactions were performed using M-MuLV-RT (Sigma-Aldrich, cat no.11785826001; Saint Louis, MO, USA) according to the manufacturer’s protocol. The total reaction volume was 10 μL. In the first step, the mixture containing 500 ng of total RNA, water, 5 mmol/μL universal oligo(dT)_10_ primer, and 300 nmol/μL random hexamer primer were denatured at 65 °C for 10 min then cooled on ice. Subsequently, 2 mmol/μL of each deoxynucleotide triphosphates, 1.5 U/reaction of *E. coli* Poly(A) Polymerase (Carolina Biosystems, cat no. PAPY-30; Prague, Czech Republic), 150 nm/μL deoxyadenosine triphosphates, 15U/reaction of ribonuclease inhibitor, 1X buffer M-MuLV-RT buffer, and 10 U/reaction of M-MuLV reverse transcriptase were added. Samples were incubated at 25 °C for 10 min, 55 °C for 60 min, then 5 min at 85 °C. Complementary DNA (cDNA) was either immediately used for quantitative polymerase chain reaction (qPCR) or stored at −20 °C until further analysis (but no longer than seven days).

#### 4.4.2. Quantitative Polymerase Chain Reaction

*ESR1* mRNA was quantified using sequence-specific primers (sense: CCTTCTTCAAGAGAAGTATTCAAGG and antisense: ATTCCCACTTCGTAGCATTTG) and the Roche Universal ProbeLibrary TaqMan^®^ hydrolysis probe (#69, cat no. 04688686001) using the ProbeFinder Assay Design Center (https://lifescience.roche.com/en_pl/brands/universal-probe-library.html, accessed on 4 October 2016). The hypoxanthine-guanine phosphoribosyltransferase (*HPRT*) gene was used as a reference gene (RealTime ready *HPRT*, Roche, cat no. 05532957001; Basel, Switzerland). The 20μL total volume reaction mixture contained 5 μL cDNA, 1X LightCycler^®^ FastStart TaqMan^®^ Probe Master (Roche, cat no. 04673417001; Basel, Switzerland), and 1X RealTime ready *HPRT* for reference gene or 200 nm of hydrolysis probe #69 along with 400 nm of the primer mixture for the gene of interest, and nuclease-free water. qPCR reactions were performed using the LightCycler^®^ 2.0 carousel glass capillary-based system (Roche). The thermal profile was performed as previously described [25]. Each sample was analyzed in duplicate with independently synthesized cDNA. The quantitative PCR results were assembled using the LightCycler Data Analysis Software version 5.0.0.38 (Roche; Basel, Switzerland), and the fluorescence measurement results were normalized to standard curves [25]. In each sample, *ESR1* expression was compared to reference gene expression in order to obtain a Cr value (concentration ratio) which corresponded to the relative *ESR1* expression level.

### 4.5. Statistical Analyses

Data analyses were performed using Statistica 13.3 software (TIBCO Software Inc.; Palo Alto, CA, USA) and PQStat 1.8.0.414 software (PQStat software; Poznan, Poland). The methylation level of specific CpG sites was analyzed in T-DMR1 and T-DMR2 separately for each CpG site in each region. The Shapiro–Wilk test was used for the normality of continuous variable distribution assessment. The differences in methylation levels between concave, convex, and superficial muscles was evaluated using repeated measures ANOVA or Friedman ANOVA with HSD Tukey and Dunn’s Bonferroni post-hoc tests, respectively. Methylation between patient subgroups with a Cobb angle ≤70° or >70° was compared using an independent t-test or Mann–Whitney U test. The correlation coefficients were determined by Pearson’s (r) or Spearman’s rank test (R). Data are presented as mean ± SE (standard error) or median with interquartiles. Data was considered statistically significant when *p* < 0.05.

## 5. Conclusions

The DNA methylation level of *ESR1* regulatory regions is specific to the muscle tissue localization in patients with idiopathic scoliosis. The lack of significant asymmetry between the concave, compared to the convex, side of the spinal curvature suggests that *ESR1* methylation level does not signify predisposition to the occurrence of IS. The difference in *ESR1* T-DMR2 CpGs methylation of the deep paravertebral muscles on the concave side of the curvature may be associated with IS severity.

## Figures and Tables

**Table 1:** Primer sequences and location.

	Primer	Sequence		Tm (°C)	GC (%)	PCR Product Size	Location with Respect to TSS	Location with Respect to ATG
ESR1 T-DMR1	→ PCR	GGGTGTATGTGAGTGTGTATGTTTAA	26	58.8	38.5	256 bp	−1107	−1341
	← PCR ^B^	ATAAAATATAACCTTTTCATACCAAACAT	29	56.8	20.7		−851	−1085
	→ SEQ	GTATGTGAGTGTGTATGTTTAAT	23	44.7	30.4	-	−1105	−1337
ESR1 T-DMR2	→ PCR	GTTTTTATTGGGTGTTATGTGTTTTGG	27	56.8	24.1	307 bp	−2886	−3120
	← PCR ^B^	AAACCTTTCCATAAATAACTCAATTAACT	29	56.8	20.7		−2579	−2813
	→ SEQ	GTTATGTGTTTTGGGAT	17	47.2	53.3	-	−2874	−3108

→ PCR—forward primer; ← PCR—reverse primer; ^B^—biotinylated primer; Tm—melting temperature, GC—guanine-cytosine content; bp—base pairs; TSS—transcription start site; ATG—start codon; → SEQ—sequencing primer.

**Table 2:** PCR mixture content and thermal profile of the reactions.

Component	Initial Concentration	Volume Added	Final Concentration	Mixture Volume
ZymoTaqTMPremix	2×	5 µL	1×	10 µL
→PCR	10 µM	1 µL	1 µM	
←PCR	10 µM	1 µL	1 µM	
DNA	100 ng/µL	0.2 µL	2 ng/µL	
Nuclease-free water		2.8 µL		
Thermal profile of the reactions				
Number of cycles	Step		Duration, temperature	
1	Initial denaturation		10 min, 95 °C	
37	Denaturation		30 s, 95 °C	
	Annealing		30 s, 54 °C	
	Extension		60 s, 72 °C	
1	Final extension		7 min, 72 °C	
1	Hold		∞, 4 °C	

→PCR—forward primer; ←PCR—reverse primer; min—minutes, s—seconds.

## Data Availability

The datasets used and analyzed during the current study are available from the corresponding author on reasonable request.

## Supplementary Materials

The following are available online at https://www.mdpi.com/article/10.3390/genes12060790/s1, Supplementary Table S1. DNA methylation level (%) within *ESR1* T-DMR1 and T-DMR2 regions in deep paravertebral muscles and superficial muscles (*n* = 29). Supplementary Table S2. DNA methylation level (%) within *ESR1* T-DMR1 and T-DMR2 regions in deep paravertebral muscles and superficial muscles in the groups of patients with Cobb angles ≤70° (*n* = 10) and >70° (*n* = 19). Supplementary Figure S1. Relative *ESR1* expression level in deep paravertebral muscles and superficial muscles. Supplementary Figure S2. Scatter plots showing correlations between *ESR1* expression and methylation level within T-DMR1 region in deep paravertebral muscles and superficial muscles. Figure S3. Scatter plots showing correlations between *ESR1* expression and methylation level within T-DMR2 region in deep paravertebral muscles and superficial muscles.

## Abbreviations

AIS: adolescent idiopathic scoliosis; cDNA: complementary DNA; ESR1: estrogen receptor 1; ESR2: estrogen receptor 2; *HPRT*: hypoxanthine-guanine phosphoribosyltransferase; IS: idiopathic scoliosis; PCR: polymerase chain reaction; PELP1: proline-, glutamic acid- and leucine-rich protein; qPCR: quantitative polymerase chain reaction; SE: standard error of mean; T-DMRs: differentially methylated regions.
