# Supplementary material for: Methylation of Estrogen Receptor 1 Gene in the Paraspinal Muscles of Girls with Idiopathic Scoliosis and Its Association with Disease Severity
Source: Genes (Basel). 2021 May 21;12(6):790. doi: 10.3390/genes12060790 (PMC8224318; doi:10.3390/genes12060790)
Supplement: Supplementary file 1 [file genes-12-00790-s001.zip › genes-1210052-supplementary.pdf]

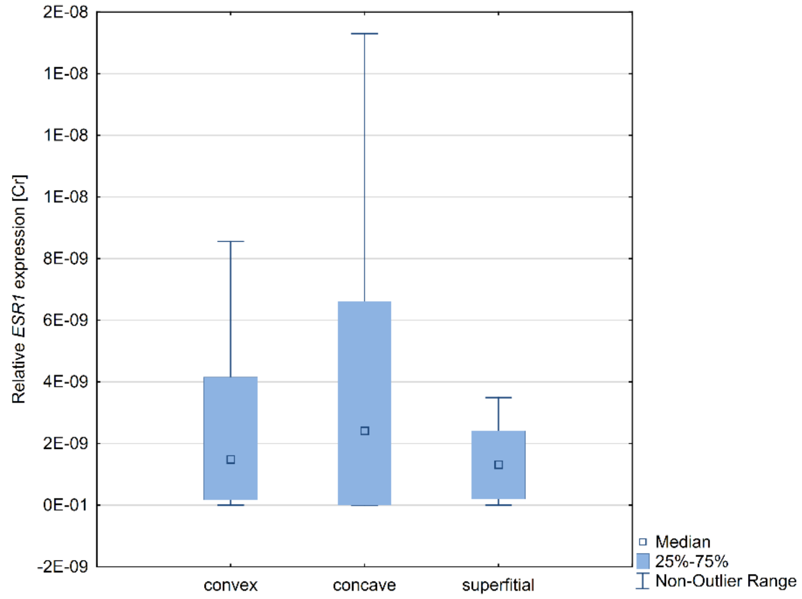

Supplementary Figure S1. Relative *ESR1* expression level in deep paravertebral muscles and superficial muscles.

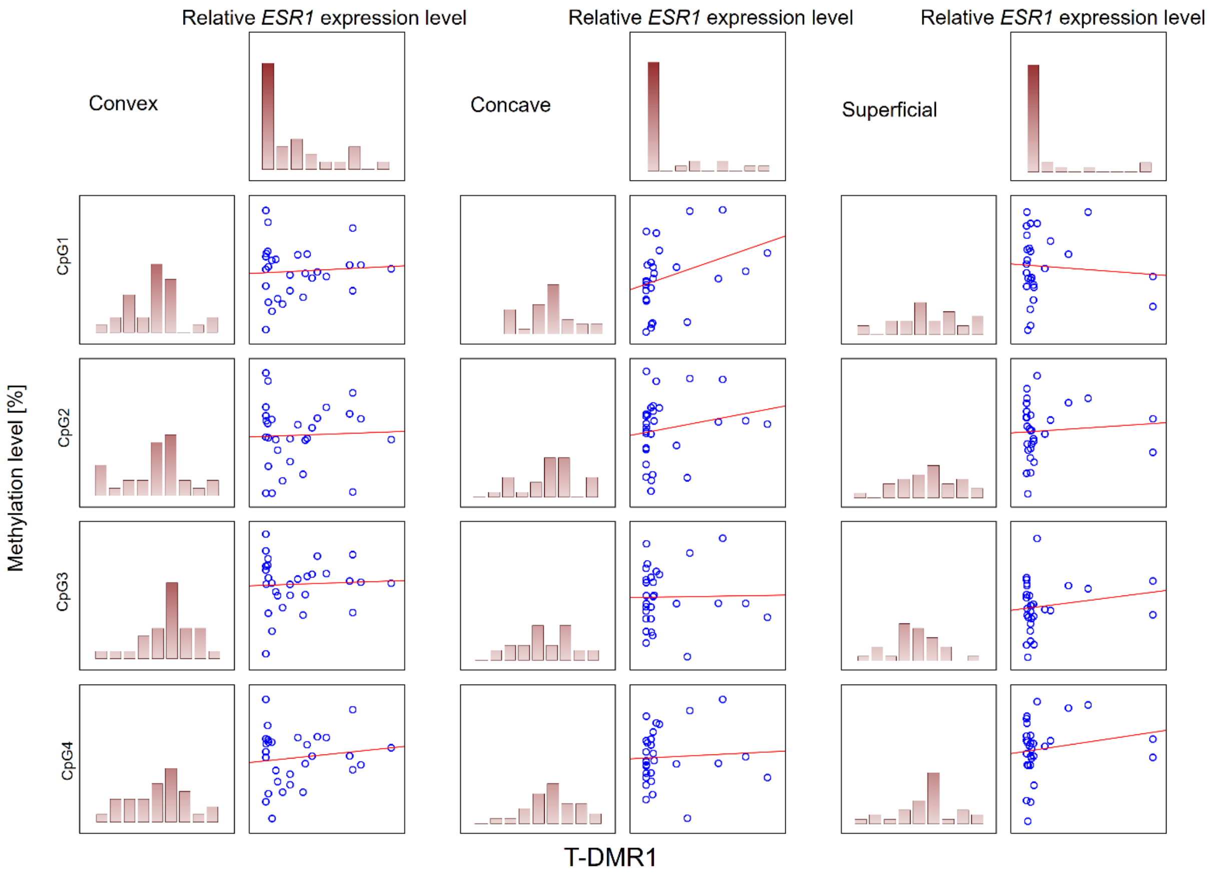

Supplementary Figure S2. Scatter plots showing correlations between *ESR1* expression and methylation level within T-DMR1 region in deep paravertebral muscles and superficial muscles.

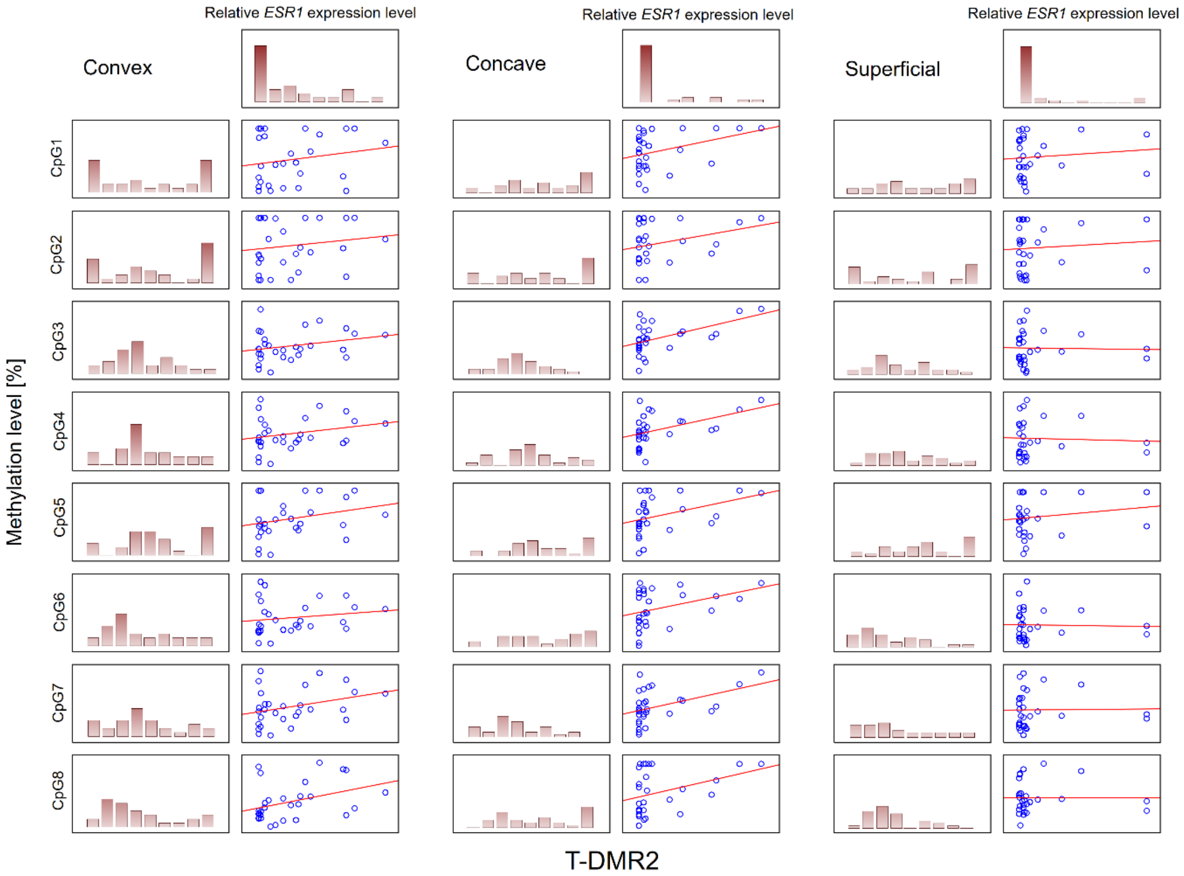

Supplementary Figure S3. Scatter plots showing correlations between *ESR1* expression and methylation level within T-DMR2 region in deep paravertebral muscles and superficial muscles.

Supplementary Table S1. DNA methylation level [%] within *ESR1* T-DMR1 and T-DMR2 regions in deep paravertebral muscles and superficial muscles (N=29).

	CpG no.	M	Me	Q1	Q3	SD	*p*-value
	**T-DMR1**						
deep muscle - convex side	CpG1	50.57	51.15	45.13	54.81	7.44	**0.0001[a]**
deep muscle - concave side		49.75	49.10	46.90	52.57	7.06	
superficial muscle		55.75	55.34	51.78	59.64	6.81	
deep muscle - convex side	CpG2	72.38	71.95	68.58	76.81	8.07	**<0.0001[a]**
deep muscle - concave side		71.26	72.85	65.60	76.54	9.50	
superficial muscle		78.73	78.95	72.59	84.26	9.51	
deep muscle - convex side	CpG3	66.84	67.62	62.78	72.16	9.33	0.11[a]
deep muscle - concave side		67.68	65.66	61.12	74.03	9.45	
superficial muscle		70.78	69.95	66.38	77.99	9.97	
deep muscle - convex side	CpG4	46.36	46.81	42.36	50.73	6.62	0.06[a]
deep muscle - concave side		47.51	46.65	43.62	51.48	6.62	
superficial muscle		49.33	50.04	47.23	52.29	6.46	
	**T-DMR2**						
deep muscle - convex side	CpG1	36.86	36.48	34.27	39.41	2.48	**0.0003[b]**
deep muscle - concave side		36.37	36.57	33.92	39.46	3.12	
superficial muscle		39.58	38.94	35.29	44.17	5.05	
deep muscle - convex side	CpG2	23.32	23.06	21.45	26.43	2.69	**0.0001[b]**
deep muscle - concave side		23.58	23.83	21.81	25.97	2.40	
superficial muscle		25.63	26.28	21.73	29.88	3.96	
deep muscle - convex side	CpG3	40.73	39.18	36.33	46.40	7.36	**0.001[b]**
deep muscle - concave side		40.38	39.98	37.07	42.95	5.47	
superficial muscle		45.31	43.63	39.91	50.13	7.81	
deep muscle - convex side	CpG4	33.88	32.22	30.38	38.42	6.92	**0.002[b]**
deep muscle - concave side		33.11	32.84	30.29	35.90	5.19	
superficial muscle		37.14	35.42	31.28	43.04	7.38	
deep muscle - convex side	CpG5	79.92	76.63	71.90	89.14	13.71	**0.03[b]**
deep muscle - concave side		82.06	79.73	74.94	91.28	12.63	
superficial muscle		85.61	84.90	76.66	100.00	10.85	
deep muscle - convex side	CpG6	22.00	20.15	18.58	25.01	4.82	**0.0004[b]**
deep muscle - concave side		21.72	21.41	19.52	24.30	3.13	
superficial muscle		24.74	23.21	19.65	28.72	6.16	
deep muscle - convex side	CpG7	44.49	43.19	38.06	52.15	9.75	**0.0007[b]**
deep muscle - concave side		44.13	42.81	40.32	47.41	7.79	
superficial muscle		48.36	45.13	40.01	55.22	10.02	
deep muscle - convex side	CpG8	15.37	13.85	11.88	17.00	5.04	**0.002[b]**
deep muscle - concave side		15.04	14.68	11.59	19.74	4.00	
superficial muscle		17.82	16.73	14.41	19.17	5.16	

[a]– ANOVA, [b] – Friedman test, M – Mean, Me – Median, Q1, Q3 – Lower and Upper Quartile, SD – Standard deviation

Supplementary Table S2. DNA methylation level [%] within *ESR1* T-DMR1 and T-DMR2 regions in deep paravertebral muscles and superficial muscles in the groups of patients with Cobb angle ≤70° (N=10) and >70° (N=19).

	**CpG no.**	**M**	**Me**	**Q1**	**Q3**	**SD**	**M**	**Me**	**Q1**	**Q3**	**SD**	***p*-value**
		Cobb angle ≤70, N=10					Cobb angle >70, N=19					
		**T-DMR1**										
deep muscle - convex side	CpG1	50.82	51.15	48.65	53.70	4.06	50.44	51.15	42.85	55.30	8.83	0.89^{c}
deep muscle - concave side		49.19	49.09	47.47	51.18	5.17	50.05	49.57	44.59	55.28	7.99	0.76^{c}
superficial muscle		53.50	52.54	47.10	59.64	7.97	56.93	56.27	53.16	61.22	6.01	0.20^{c}
deep muscle - convex side	CpG2	73.48	74.87	71.82	76.46	4.49	71.80	71.36	64.53	79.54	9.50	0.51^{d}
deep muscle - concave side		72.95	74.35	69.69	76.54	8.16	70.37	72.68	60.72	77.39	10.23	0.50^{c}
superficial muscle		77.37	77.46	71.24	83.24	10.57	79.45	80.40	72.59	85.54	9.13	0.58^{c}
deep muscle - convex side	CpG3	68.76	68.64	66.88	72.16	5.77	65.82	67.28	58.18	73.62	10.75	0.43^{c}
deep muscle - concave side		68.62	66.75	64.50	73.27	8.54	67.18	65.61	60.63	74.97	10.08	0.70^{c}
superficial muscle		70.51	71.40	66.78	79.17	9.77	70.93	69.59	65.93	77.99	10.34	0.92^{c}
deep muscle - convex side	CpG4	47.61	48.28	46.41	50.19	4.69	45.71	44.93	39.86	50.95	7.48	0.47^{c}
deep muscle - concave side		48.93	48.62	46.65	51.48	5.01	46.76	45.60	42.52	51.85	7.35	0.41^{c}
superficial muscle		48.80	49.70	47.68	51.57	6.56	49.62	50.64	45.70	52.41	6.57	0.75^{c}
		**T-DMR2**										
deep muscle - convex side	CpG1	36.89	36.54	35.22	39.06	2.08	36.85	36.36	34.03	39.99	2.72	0.95^{d}
deep muscle - concave side		35.06	35.00	33.48	36.71	2.87	37.06	38.10	33.97	40.13	3.09	0.11^{d}
superficial muscle		38.09	38.00	34.64	41.92	5.11	40.37	40.02	37.08	45.24	4.97	0.25^{d}
deep muscle - convex side	CpG2	22.94	22.51	21.87	24.93	2.38	23.51	24.04	20.37	26.43	2.88	0.67^{d}
deep muscle - concave side		22.16	22.13	19.50	23.91	2.25	24.32	24.13	22.45	26.40	2.17	**0.02^{d}**
superficial muscle		24.55	25.59	20.87	26.85	3.53	26.20	26.61	22.83	30.57	4.15	0.33^{d}
deep muscle - convex side	CpG3	39.60	39.04	36.67	40.95	5.47	41.33	39.37	35.19	47.94	8.25	0.56^{c}
deep muscle - concave side		37.98	38.15	36.05	39.89	5.90	41.65	42.03	37.95	44.59	4.93	**0.04^{d}**
superficial muscle		42.64	40.30	37.65	49.45	7.11	46.72	44.50	41.04	52.30	7.97	0.19^{c}
deep muscle - convex side	CpG4	31.14	30.39	30.05	32.43	4.76	35.32	33.77	31.12	40.27	7.53	0.12^{c}
deep muscle - concave side		30.37	30.82	26.72	32.84	4.97	34.55	34.09	31.59	37.78	4.81	**0.04^{c}**
superficial muscle		33.86	31.41	29.67	39.17	5.79	38.87	35.57	31.92	46.40	7.66	0.08^{c}
deep muscle - convex side	CpG5	77.98	75.91	73.64	84.70	11.17	80.94	79.73	71.55	100.00	15.06	0.60^{c}
deep muscle - concave side		82.03	78.49	76.34	85.77	10.96	82.07	81.85	72.79	95.32	13.72	0.99^{c}
superficial muscle		83.49	83.48	75.59	87.26	10.69	86.73	85.74	76.66	100.00	11.06	0.46^{c}
deep muscle - convex side	CpG6	20.05	19.83	18.58	20.84	2.89	23.03	21.45	18.47	28.64	5.35	0.11^{c}
deep muscle - concave side		19.57	19.65	18.57	20.90	2.11	22.86	23.99	21.01	25.03	3.00	**0.005^{d}**
superficial muscle		21.79	20.40	18.78	24.79	4.04	26.29	24.42	20.98	30.75	6.60	0.06^{c}
deep muscle - convex side	CpG7	41.12	41.47	38.06	43.32	7.24	46.26	45.52	37.55	52.94	10.58	0.18^{c}
deep muscle - concave side		40.58	40.87	36.00	42.45	6.61	45.99	45.76	40.70	52.38	7.87	0.07^{c}
superficial muscle		44.44	41.79	38.19	49.79	7.26	50.42	46.04	41.18	60.70	10.80	0.13^{c}
deep muscle - convex side	CpG8	14.45	12.34	11.04	16.90	4.75	15.86	14.81	11.88	17.99	5.24	0.37^{d}
deep muscle - concave side		13.94	11.83	11.00	17.97	4.02	15.62	15.71	11.59	20.40	3.97	0.35^{d}
superficial muscle		16.10	16.71	12.78	17.93	4.60	18.73	17.24	15.09	24.41	5.32	0.29^{d}

^{c} –t-test, ^{d} - U-Mann-Whitney test, M – Mean, Me – Median, Q1, Q3 – Lower and Upper Quartile, SD – Standard deviation
